# Corneal cross-linking in 9 horses with ulcerative keratitis

**DOI:** 10.1186/1746-6148-9-128

**Published:** 2013-06-26

**Authors:** Anna Hellander-Edman, Karim Makdoumi, Jes Mortensen, Björn Ekesten

**Affiliations:** 1 Department of Animal Environment & Health, Swedish University of Agricultural Sciences, PO Box 234, SE-532 24 Skara, Sweden; 2 Department of Ophthalmology, Örebro University Hospital, SE-701 85 Örebro, Sweden; 3 Department of Clinical Sciences, Swedish University of Agricultural Sciences, PO Box 7054, SE-750 07 Uppsala, Sweden

**Keywords:** Equine, Horse, Keratitis, Corneal ulceration, Cross-linking, Cross linking, Collagen, CXL, UVA, Riboflavin, Stromal melting

## Abstract

**Background:**

Corneal ulcers are one of the most common eye problems in the horse and can cause varying degrees of visual impairment. Secondary infection and protease activity causing melting of the corneal stroma are always concerns in patients with corneal ulcers. Corneal collagen cross-linking (CXL), induced by illumination of the corneal stroma with ultraviolet light (UVA) after instillation of riboflavin (vitamin B2) eye drops, introduces crosslinks which stabilize melting corneas, and has been used to successfully treat infectious ulcerative keratitis in human patients. Therefore we decided to study if CXL can be performed in sedated, standing horses with ulcerative keratitis with or without stromal melting.

**Results:**

Nine horses, aged 1 month to 16 years (median 5 years) were treated with a combination of CXL and medical therapy. Two horses were diagnosed with mycotic, 5 with bacterial and 2 with aseptic ulcerative keratitis. A modified Dresden-protocol for CXL could readily be performed in all 9 horses after sedation. Stromal melting, diagnosed in 4 horses, stopped within 24 h. Eight of nine eyes became fluorescein negative in 13.5 days (median time; range 4–26 days) days after CXL. One horse developed a bacterial conjunctivitis the day after CXL, which was successfully treated with topical antibiotics. One horse with fungal ulcerative keratitis and severe uveitis was enucleated 4 days after treatment due to panophthalmitis.

**Conclusions:**

CXL can be performed in standing, sedated horses. We did not observe any deleterious effects attributed to riboflavin or UVA irradiation per se during the follow-up, neither in horses with infectious nor aseptic ulcerative keratitis. These data support that CXL can be performed in the standing horse, but further studies are required to compare CXL to conventional medical treatment in equine keratitis and to optimize the CXL protocol in this species.

## Background

Corneal ulcers are one of the most common eye problems in the horse and can cause varying degrees of visual impairment or even blindness [1,2]. Secondary infection and protease activity causing melting of the corneal stroma are always concerns for the clinician, even in horses with superficial ulcers. Exposure of a corneal ulcer to organisms from both the ocular surface and the environment may result in secondary infection, which combined with infiltrating polymorphonuclear leukocytes, stimulates production and activity of proteolytic enzymes and subsequent corneal melting [1,3,4]. Conventional medical treatment of the melting process involves controlling infection with antibiotics and managing pain. However, antibiotic treatment of equine corneal ulcers can lead to acquired antibiotic resistance and selective propagation of resistant bacteria [5,6].

The term cross-linking refers to the formation of covalent bonds between long chained molecules. Intermolecular cross-linking increases the rigidity of materials and is also a natural part in the normal aging of the corneal stroma [7,8]. Corneal cross-linking (CXL) is induced by introducing riboflavin (vitamin B2) to the cornea and illuminating it with ultraviolet light (UVA). Riboflavin absorbs UVA and visible light, producing reactive oxygen species (ROS) [9]. In the cornea, ROS increase the number of covalent bonds among stromal collagen molecules [10], which enhances the biomechanical rigidity of the cornea [11-14]. Furthermore, CXL changes molecular structures within the cornea to prevent proteolytic enzymes from binding to specific cleavage sites, decreasing the efficacy of collagen degrading enzymes [15]. Thus, through increasing biomechanical strength and decreasing proteolysis, CXL has been demonstrated to be effective in cases of keratoconus and melting ulcers in humans [16,17].

Riboflavin has been used for inactivation of pathogens in blood products [18], due to its ability to induce strand cleavage of DNA and RNA, thus interfering with the replication of pathogenic microorganisms [19,20]. In its application to the cornea, CXL has been documented to exert antimicrobial effects, both in vitro [21,22] and in vivo [23-26]. The specific mechanisms remain to be elucidated, but it is possible that the increased resistance of collagen to degradative enzymes, as well as a direct antimicrobial effect of ROS may be involved [27].

In human eyes, cross-linking of the cornea appears to be a safe procedure. However, two potential risks to the eye from UVA exposure have to be considered: direct damage to corneal cells and intraocular structures from UVA irradiation, and indirect damage to all regional cells from ROS [28]. Riboflavin limits the potential for direct damage, as it limits radiant transmission to deeper ocular structures by absorbing UVA-light. In human, porcine, and rabbit corneas, damage to keratocytes occurs to a depth of 300 μm with surface irradiance of 3 mW/cm^2^[29-31], thus it is recommended that CXL only be conducted when corneal thickness exceeds 400 μm. This provides a margin of safety that avoids direct damage to the corneal endothelium and intraocular structures [32,33]. The loss of stromal keratocytes in the illuminated area can be accepted, because it is temporary to current knowledge and repopulation occurs during 6 months after the UVA illumination [34,35].

Since stromal ulcerative keratitis is a common condition in the horse, frequently associated with secondary infection and melting of the stroma, we decided to test whether it was possible to perform CXL in sedated, standing horses. We also wanted to know if equine stromal ulcerative keratitis with and without melting would heal after treatment with a combination of CXL and medical therapy.

## Results

All horses were diagnosed with stromal ulcerative keratitis with leukocyte infiltrates surrounding the ulcers (Table 1). Four horses showed clinical signs of corneal melting. Microbiological cultures in 7 out of 9 horses were positive for bacteria or fungi.

**Table 1:** Details of 9 horses treated with CXL and medical treatment for stromal ulcerative keratitis

**Horse**	**Breed**	**Sex and age**	**Disease history**	**Topical medication pre CXL**	**Stromal ulcer size; depth pre CXL**	**Stromal melting**	**Comments**	**Microbes on cytology and in culture pre CXL**	**Fluor. neg. post CXL**	**Follow up time**
1	Mixed breed pony	G 6 yr	4 w	Fucidic acid 1 w, chloramphenicol 2 w, natamycin 1 w	5 × 5 mm; 1/3	no	Hypopyon	Abundant fungal hyphae/ Yeast overgrowth, mixed bacteria	Enucl.	n/a
2	Swedish Warmblood	G 5 yr	4½ w	Fucidic acid 1 w, chloramphenicol-ciprofloxacin 2½ w, natamycin 1 w	4 × 4 mm; 1/3 7 × 7 mm; 1/3	yes		Abundant fungal hyphae/ Scedosporium spp.	9 d	19 mo
3	Warmblood Trotter	F 1 yr	1 w	Chloramphenicol 1 w	10 × 10 mm; 2/3	yes	Extensive cellular infiltration. Severe purulent discharge	Abundant coccoid bacteria/ ß-hem. Streptococcus	20 d	12 mo
4	Islandic horse	S 2 yr	< 1 w	Chloramphenicol < 1w	8 × 8 mm; 2/3	no	Hypopyon	Sparse coccoid bacteria/ ß-hem. Streptococcus	9 d	11 mo
5	Mixed breed pony	F 5 yr	> 2 w	Fucidic acid >1 w, chloramphenicol + autologous serum in EDTA 1 w	4 × 4 mm; 1/3 5 × 5 mm; 2/3	no	Two stromal ulcers in large superficial ulcer. Severe purulent discharge	No bacteria/ No growth	9 d + 16 d	9 mo
6	Miniatur Shetland Pony	F 16 y	< 1 w	Fucidic acid < 1 w	8 × 8 mm; 2/3	no	Stromal ulcer in large superficial ulcer	Abundant bacteria/ Untyped G + bacteria	14 d	6 mo
7	Warmblood Trotter	F 1 mo	< 1 w	No	10 × 13 mm; 2/3	yes	Extensive cellular infiltration. Severe purulent discharge	Abundant bacteria/ Pasteurella spp	4 d	5 mo
8	Welsh Pony	S 2 mo	1 w	Chloramphenicol 1 w	5 × 5 mm; 2/3	no		Moderate bacteria/ Untyped G + coccoid	14 d	5 mo
9	Welsh Pony	F 15 yr	1 d	No	6 × 18 mm; 1/3	yes	Corneal laceration	No bacteria/ No growth	26 d	5 mo

F = female (mare), G = gelding, S = stallion, d = days, mo = months, w = weeks, yr = years, 1/3 = anterior third of the cornea, 2/3 = middle third of the cornea, G + =Gram-positive, ß-hem = ß-hemolytic, enucl = enucleated, n/a = not applicable. All horses were affected unilaterally. For horse no 5, the number of days to fluorescein negativity is listed for each of the two stromal ulcers respectively.

CXL could be performed in all 9 horses. Removal of a narrow zone of the surrounding corneal epithelium, instillation of riboflavin eye drops and exposure of the ulcerated area to UVA light was well tolerated by the sedated horses. Each treatment session, from sedation to completion of the CXL (including sampling for cytology and microbiology), took approximately two hours. Instillation of riboflavin can readily be performed by one person, whereas other parts of the procedure require both a veterinarian and a nurse.

**Figure 1:**
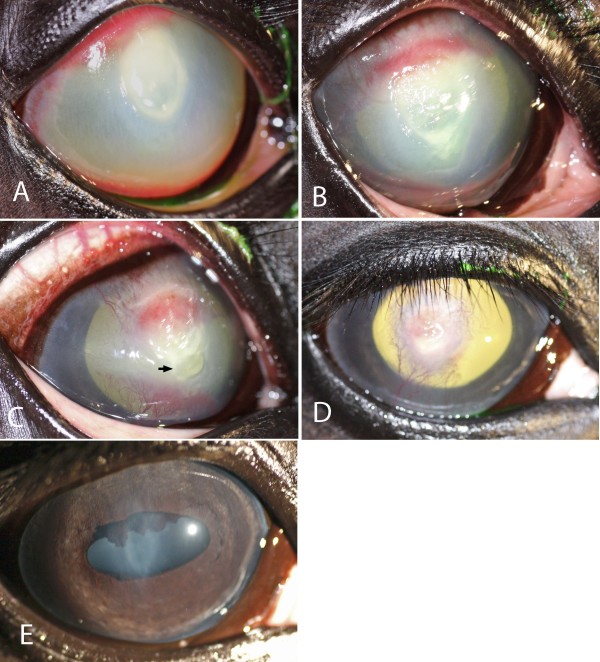
**The healing process during follow-up after CXL. A**) Horse no. 3 before CXL. This horse had been on topical antibiotic treatment for a stromal ulcer infected by ß-hemolytic streptococci for a week before being referred for CXL. Panels **B**) to **E**) show the appearance of the cornea from 5 days up to 12 months after CXL in combination with topical and systemic medication (**B**) = 5, **C**) = 13, **D**) = 20 days and **E**) = 12 months). Notice the formation and rejection of necrotic material (arrow) in the ventral part of the ulcer at 13 days. Scarring is minimal a year after treatment.

**Figure 2:**
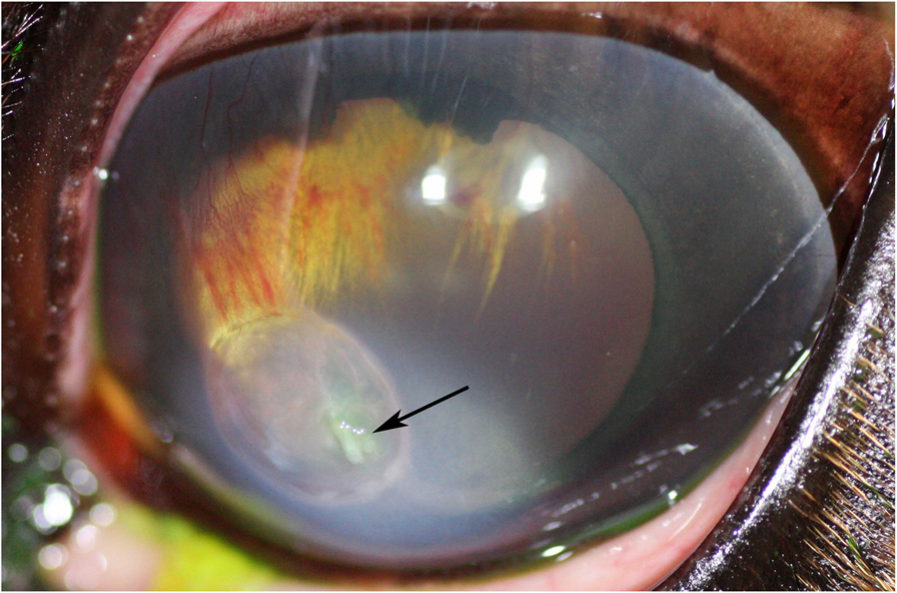
**Rejection of necrotic tissue.** Rejection of necrotic tissue during healing of the ulcer after 6 days in horse no. 5. The rejection process did not appear to cause discomfort for the patient.

Eight of nine eyes treated with CXL combined with topical and systemic medical treatment (as described in the Methods section), healed with light stromal scarring (Figure 1). Within 3 days, all of these eight eyes showed improvement of clinical signs. The healing process of the stromal ulcers after CXL followed a similar pattern with respect to the reduction of inflammatory signs, including reduced signs of ocular pain, decrease in corneal oedema and ciliary injection and less aqueous flare and inflammatory cells in the anterior chamber. The stromal melting had stopped when re-examined the day after treatment. In 2–4 days, a granular appearance of the denuded stromal tissue could be observed and in some cases a rejection of necrotic mucoid tissues followed (Figure 2). Ingrowth of blood vessels to the ulcerated area and regeneration of the corneal stroma and re-epithelialization could be observed. Eight of nine eyes became fluorescein negative in 13.5 days (median time; range 4–26 days) after CXL (Table 2).

**Table 2:** Median healing time of stromal ulcers

	**CLX – fluorescein neg. [days]**	**Onset of clinical signs – fluorescein neg. [days]**
Horses with and without positive microbial culture (n = 8)	13.5 (4–26)	22 (10–40)
Horses with positive microbial culture (n = 6)	11 (4–20)	22 (10–40)

The median healing time with ranges. Horse no. 1, which was enucleated, is excluded from the table.

CXL treatment failed in one of the two horses with fungal ulcerative keratitis (Table 1). This horse, horse no. 1, developed panophthalmitis during 4 days after the CXL and the eye was enucleated. Histopathology of the eye showed keratomalacia with ulcerations of Descemet’s membrane and infiltration of fungal hyphae into the anterior chamber.

**Figure 3:**
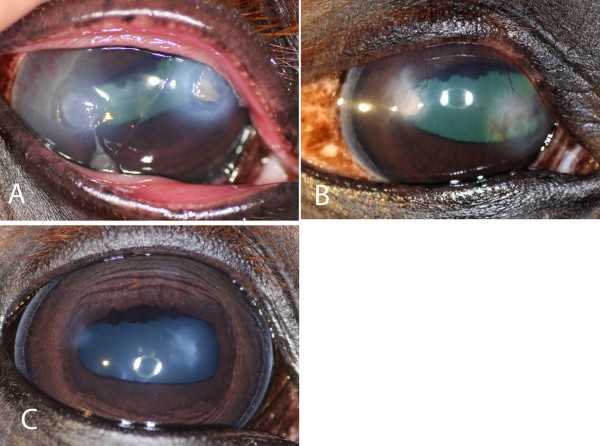
**The healing process and follow-up of fungal ulcers after CXL. A**) Horse no. 2 presented with 2 stromal ulcers that had been treated for 1 month, initially with topical antibiotics and during the last week before CXL, when a diagnosis of fungal ulcerative keratitis was made, with topical natamycin. However, the response was considered poor and CXL was performed. The cornea was fluorescein negative at 9 days after CXL. **B**) The appearance 30 days and **C**) 19 months after CXL.

In horse no. 2, neither fungal hyphae nor bacteria were observed on cytology four days after CXL. The two stromal ulcers in this horse became fluorescein negative in 5 and 9 days after CXL, respectively (Figure 3).

Horse no. 3, which had streptococcal keratitis, was negative on corneal culture and no bacteria were present on cytology 5 days after CXL, despite the lack of topical antimicrobial treatment after CXL (Figure 1).

One patient, horse no. 2, developed purulent conjunctivitis the day after CXL treatment. Bacterial culture from the conjunctiva was positive for Enterobacter and the conjunctivitis resolved after topical treatment with chloramphenicol.

## Discussion

CXL could readily be performed in all 9 horses under sedation and standing surgery. The procedure is rather time-consuming to perform compared to medical treatment alone, but easy to learn and does not require expertise in microsurgery. However, evaluation of pre- and postoperative results should be performed by a person with proper training in equine ophthalmology. Because of the risks associated with general anaesthesia in this species, it is an advantage to be able to perform the treatment on standing horses under sedation. A stable level of sedation and a calm environment are crucial for proper positioning of the UVA light on the cornea of the horse throughout the procedure.

The melting process seen in four horses had stopped when re-examined the day after treatment. This is in accordance with the results in a pilot study where CXL was used to treat three cats and two dogs with melting corneal ulcers [36], as well as in human patients [17]. CXL is believed to increase the resistance of the cornea to collagen digesting enzymes, because of the structural changes of the collagen fibrils induced by cross-linking [15,37]. In the horse, melting of the corneal stroma is not uncommon in patients with corneal ulcers. Frequent instillation of eye drops onto a rapidly melting cornea and painful eye can be rather challenging and certainly time-consuming. The prompt increase in stability of the melting cornea seen in various species suggests that CXL could be beneficial and labour-saving in patients with uncontrolled enzymatic degradation of the corneal stroma.

A few days after CXL we observed that the denuded stroma took on a granular appearance in most eyes. This has not been described in other species and might reflect a phase during the post-CXL wound healing that may be specific to the horse. In three horses this was followed by formation of a tissue layer with mucoid, necrotic appearance that was rejected after approximately 1–2 weeks.

Corneal ulcers in the horse often require medical treatment for several weeks and sometimes adjunct corneal surgery [38]. In our study, the median time from CXL to fluorescein negativity was 11 days in horses with positive microbial cultures. Ulcerative keratitis caused by Streptococci is frequently reported to be refractory to treatment, producing stromal melting. Brooks et al. reported an average healing time of 44.7 ± 26,7 days in horses with streptococci isolated from corneal ulcers and the vast majority of the horses required corneal surgery [39]. Andrew et al. reported a median duration of treatment of 48 days for 29 horses with ulcerative keratomycosis [40]. In a study by Wada and colleagues, the mean healing time was 38.9 ± 24.0 days in 26 horses with positive microbial cultures [2]. Our median healing time is closer to the median time for re-epithelialization reported in human patients with bacterial keratitis primarily treated with CXL (5.5 days; range 1–14 days) [26]. Because of the low number of horses in this study and the absence of a control group, we cannot conclude that the healing time after CXL is different from the time to healing in horses treated medically only.

The CXL was unsuccessful in horse number 1 with fungal keratitis. Light microscopic examination of the globe enucleated four days postoperatively revealed fungal hyphae inside the anterior chamber. At the day of CXL-treatment, but before CXL was performed, this eye showed an aggravation of clinical signs, including hypopyon, pain and miosis. Perhaps the fungi in this case had spread intraocularly already before treatment with CXL. It is also possible that the antifungal effect of the CXL was insufficient, as has been indicated by the work of Martins et al. [22]. Because the antimicrobial effect of CXL is restricted to the superficial 300 μm using this type of UVA light source, it is unlikely that microorganisms dwelling deep in the corneal stroma, would be completely eliminated. Consequently, this case shows that the indications for CXL in the horses with stromal ulcerative keratitis need to be carefully considered.

We experienced one patient that developed a purulent conjunctivitis due to Enterobacter sp. the day after CXL. Enterobacter is known to be an opportunistic species of bacteria not uncommonly isolated from horses with ulcerative keratitis and conjunctivitis [2,41]. It is likely that the manipulation of the eye during the examination or treatment of the ulcer played a role in the development of the bacterial conjunctivitis in this horse, but we do not believe that the exposure to the UVA-light or riboflavin eye drops per se caused this problem. Hence, measures to reduce the risk for iatrogenic infections should, as always, be taken and careful postoperative monitoring of the outcome is recommended.

Potential complications of CXL in patients include loss of endothelial cells due to incomplete absorption of the UVA light in the overlying stroma, as well as loss of limbal stem cells after accidental illumination by the UVA light. It has been shown experimentally that CXL of thin corneas or of corneas with insufficient concentration of riboflavin, for instance due to too short exposure to the riboflavin eye drops to allow sufficient absorption, can cause endothelial cell damage [28,32,42]. The normal equine cornea is 0.8—1.0 mm thick [1], therefore, with exception of very deep corneal ulcers, the risk for endothelial damage is judged to be low. To reduce the risk for uncontrolled UV damage to the cornea, we used a light source with an emission spectrum coinciding with the peak absorption of riboflavin in the UVA-part of the spectrum (365 nm) [43], an optical system providing a rather constant beam intensity across the area illuminated to prevent hot spots and a shutter that allows light-exposure of a well defined area of tissue to avoid incidental damage to limbal stem cells. Furthermore, the radiant exposure and the irradiance used were below the threshold for damaging the human eye by UVA [28,44].

Makdoumi et al., who treated human patients with mainly infectious, ulcerative keratitis, a patient cohort rather similar to our horses, observed no complications that could be attributed to CXL up to 6 months after treatment [26]. We have not observed chronic corneal oedema, which would have been a clinical sign of endothelial decompensation, during the follow up period up to 19 months. Furthermore, light corneal scarring has been reported in a low number of patients with keratoconus treated with CXL [45-47]. Postoperative scarring was present but limited in the 8 horses where CXL was successful in this study and all these horses retained useful vision. Bearing the pre-operative appearance of these corneas, we were impressed with the corneal transparency months after CXL, but again it is not possible to judge whether the CXL treatment has affected the amount of scarring seen postoperatively. However, corneal scarring is considered a problem in equine infectious keratitis no matter whether surgery is performed or not [39].

In summary, vision-threatening complications directly associated with the procedure were not observed in this pilot study. We believe that further studies on CXL in this species are warranted.

## Conclusions

CXL-treatment was readily performed in 9 sedated, standing horses. The median duration from CXL to fluorescein negativity was 13.5 days in the 8 horses where the corneal ulcers healed. Stromal melting seen in 4 horses stopped within 24 h after CXL. Postoperative corneal scarring was subjectively judged to be light, but comparison to similar ulcers only treated medically was not available. One patient contracted a postoperative, bacterial conjunctivitis and CXL showed no therapeutic effect in one horse. This shows that patients eligible for CXL need to be carefully chosen and monitored postoperatively. We consider CXL potentially useful for treatment of stromal corneal ulcers in the horse, but further research and comparison to conventional medical treatment is needed.

## Methods

### Animals

Nine horses, aged 1 month to 16 years (median 5 years) of different breeds and gender were treated for stromal ulcerative keratitis with CXL using riboflavin and UVA-light during 2010 and 2011 at ATG’s Horse Clinic in Skara, Sweden (Table 1). All horses, except for 2 previously untreated horses, were referred because of failure of medical therapy, defined as non-healing ulceration or aggravation of clinical signs from the eye, specifically increased corneal vascularization, oedema, or stromal loss, or emergence of additional ulcers. The two previously untreated horses were included in the study upon request from the owners.

The regional Ethical Committee (Göteborgs Djurförsöksetiska nämnd, Sweden, Dnr 246–2010) approved the use of horses for this study and informed consent was obtained from all horse owners prior to treatment.

### Ophthalmic examination

All horses were examined by the first author before and after the CXL treatment. The horses were sedated and akinesia of the eyelids was induced when needed. The ophthalmic examination included slit-lamp biomicroscopy (Kowa SL15, Kowa Company Ltd, Nagoya, Japan), sampling for cytology and microbiological testing, as well as fluorescein staining. Samples for microbiological cultures and cytology were obtained from the edge of the ulcerations using a sterile swab (ESwab, Copan innovation Ltd, Brescia, Italy) and a special brush (Cytology brush Plus, CooperSurgical Inc., Trumbull, USA), respectively. Aerobic bacterial culture and antimicrobial susceptibility testing was performed by a local laboratory (Eurofins, Lidköping, Sweden). Specimens for cytology were stained using May Grünwald Giemsa and Gram staining, respectively. When fungal hyphae were present on cytology, an additional swab was sent for fungal culture. Microbiological cultures and cytology were repeated after CXL in horses nos. 2 and 3 to learn if the microorganisms obtained in the initial samples were eliminated after treatment. All horses were re-examined until the ulcers healed, defined as no fluorescein dye uptake, and absence of clinical signs of pain or anterior uveitis. Photographs of the corneas were taken before CXL and at each re-examination.

The depths of the ulcers were scored according to Burn's system [48]. Only horses with stromal loss comprising ≤ 2/3 of the corneal thickness were included in the study.

The diagnosis of stromal melting was made when the stroma had a gelatinous structure and an abnormal, often drop-shaped contour. When the progressive stromal melting ceased, the structure stabilized and the size of the ulcer stopped increasing.

### Sedation, local and topical anesthesia

The adult horses were sedated with 0.01 mg/kg detomidine (Domodin vet, 10 mg/ml, Novartis Animal Health, Copenhagen, Denmark) and 0.02 mg/kg buthorphanol (Butador vet 10 mg/ml, Vetoquinol Scandinavia, Åstorp, Sweden) intravenously. Sedation was maintained throughout the procedure using symptomatic i.v. infusion with 1 mg detomidine per 100 ml physiologic saline solution (Natriumklorid, Fresenius Kabi, Uppsala, Sweden).

The two foals were anesthetized with a combination of 1.0 mg/kg xylazine (Rompun 20 mg/ml, Bayer Animal Health, Copenhagen, Denmark), 0.05 mg/kg midazolam (Midazolam 5 mg/ml, Actavis AB, Stockholm, Sweden) and 2.0 mg/kg ketamine (Ketaminol vet, 100 mg/ml, Intervet AB, Sollentuna, Sweden).

Akinesia of the eyelids was obtained after subcutaneous injection of 1 ml mepivacaine (Carbocain, 20 mg/ml, Astra Zeneca, Södertälje, Sweden) over the auriculopalpebral nerve. The cornea was anesthetized with 2 drops of oxybuprocaine eye drops (Oxibuprokain Chauvin, 20 mg/ml, Bausch & Lomb Nordic AB, Stockholm, Sweden).

### Corneal collagen cross-linking, CXL

The CXL treatment protocol was based on the protocol developed in Dresden and described by Wollensak et al. in 2003 [33], with minor modifications for horses. Two to 3 mm of the epithelium surrounding the corneal ulcer was removed using a blunted scalpel blade. Isotonic riboflavin eye drops (Medio-cross®, riboflavin > 0.1 in dextran 500 20%, Medio-cross GmbH, Neudorf, Germany) were instilled every 2^nd^ minute for 25 minutes. The non-epithelialized part of the cornea was saturated with riboflavin, which could be observed as a yellowish discoloration.

**Figure 4:**
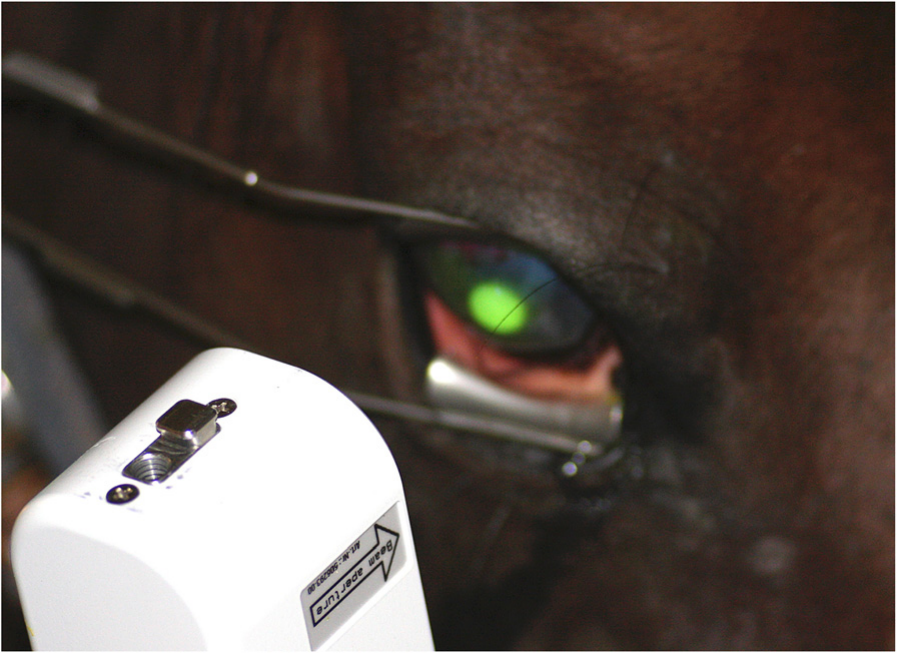
**Corneal collagen cross-linking.** A handheld UVA-light source was employed to irradiate the corneal ulcer during 30 minutes. The edge of the light beam was sharply circumscribed when the lamp was held at a distance of 5 cm from the corneal surface, which enhanced maintaining the correct distance between the lamp and the patient’s eye.

A lid speculum was inserted to keep the eye open during light exposure. Prior to each treatment, the UVA-light source (CCL-365, Peschke Meditrade GmbH, Huenenberg, Switzerland) was calibrated according to the manufacturer’s recommendations. The corneal ulcer was then exposed during 30 minutes to a 365 nm light with an irradiance of 3.0 mW/cm^2^ and a beam diameter of 10 mm (Figure 4). This light source has an optical system that provides a uniform (Köhler) illumination of the treated area [49]. The distance between the UVA-light source and the cornea (5 cm) was maintained throughout the procedure by supporting the light source by one hand resting against the head of the horse. The light source would then also follow any movements made by the horse and stay centred on the ulcerated area. The operator continuously monitored that the beam remained focused and sharply circumscribed at the corneal surface. Care was taken not to illuminate the limbus with the UV-light.

All horses except horse no. 3 received topical medication after CXL treatment, according to a standard protocol, with 5 ml chloramphenicol eye drops (Chloromycetin, 5 mg/ml, Pfizer AB, Sollentuna, Sweden) and 1 ml atropine eye drops (Isopto-Atropin 1%, Alcon Sverige AB, Stockholm, Sweden) diluted in autologous serum in 1.8 mg/ml EDTA (serum collected in EDTA-tubes) to a total volume of 10 ml. This mixture was instilled on the eye through a continuous delivery system (Infu-Disc™, San Diego, CA, USA) at a rate of 0.14 ml/h. Accidently, horse no. 3 got autologous serum and atropine only in the delivery system. To relieve postoperative discomfort and treat concomitant uveitis, 1.1 mg/kg flunixin meglumine (Cronyxin vet 50 mg/ml, Ceva Animal Health, Lund, Sweden) was given intravenously once daily for 5–8 days in all horses. Horse nos. 3, 7 and 8 were also given penicillin G sodium (Geepenil vet 300 mg/ml, Orion Pharma Animal Health, Sollentuna, Sweden) 14 mg/kg, intravenously q8h, for 6, 8 and 4 days respectively.

### Statistical analyses

Medians and ranges are used as descriptive statistics in this study.

## Competing interests

The authors declare that they have no competing interests.

## Authors’ contributions

AHE conceived the study, performed ophthalmic examinations and CXL treatments, analysed the data and helped to draft the manuscript. KM provided advice regarding the CXL protocol and helped to draft manuscript. JM proposed the CXL protocol and assisted when CXL was performed. BE helped with the study design, analysis of results and to draft the manuscript. All authors read and approved the final manuscript.

## References

[B1] Brooks DE Matthews AG Gelatt KN Equine ophthalmology Veterinary ophthalmology. Volume II 2007 4 Ames, IA, USA: Blackwell Publishing Professional 1192 1211

[B2] Wada S Hobo S Niwa H Ulcerative keratitis in thoroughbred racehorses in Japan from 1997 to 2008 Vet Ophthalmol 2010 13 2 99 105 10.1111/j.1463-5224.2010.00767.x 20447028

[B3] Johns IC Baxter K Booler H Hicks C Menzies-Gow N Conjunctival bacterial and fungal flora in healthy horses in the UK Vet Ophthalmol 2011 14 3 195 199 10.1111/j.1463-5224.2010.00867.x 21521444

[B4] Clode AC Matthews AG Gilger BC Diseases and surgery of the cornea Equine ophthalmology 2011 2 Maryland Heights, MO, USA: Elsevier Saunders 181 215

[B5] Moore CP Collins BK Fales WH Antibacterial susceptibility patterns for microbial isolates associated with infectious keratitis in horses - 63 cases (1986–1994) J Am Vet Med Assoc 1995 207 7 928 933 7559027

[B6] Sauer P Andrew SE Lassaline M Gelatt KN Denis HM Changes in antibiotic resistance in equine bacterial ulcerative keratitis (1991–2000): 65 horses Vet Ophthalmol 2003 6 4 309 313 10.1111/j.1463-5224.2003.00312.x 14641828

[B7] Cannon DJ Davison PF Aging, and crosslinking in mammalian collagen Exp Aging Res 1977 3 2 87 105 10.1080/03610737708257091 885155

[B8] Malik NS Moss SJ Ahmed N Furth AJ Wall RS Meek KM Ageing of the human corneal stroma: structural and biochemical changes Biochim Biophys Acta 1992 1138 3 222 228 10.1016/0925-4439(92)90041-K 1547284

[B9] Baier J Maisch T Maier M Engel E Landthaler M Bäumler W Singlet oxygen generation by UVA light exposure of endogenous photosensitizers Biophys J 2006 91 4 1452 1459 10.1529/biophysj.106.082388 16751234 PMC1518628

[B10] McCall AS Kraft S Edelhauser HF Kidder GW Lundquist RR Bradshaw HE Dedeic Z Dionne MJC Clement EM Conrad GW Mechanisms of corneal tissue cross-linking in response to treatment with topical riboflavin and long-wavelength ultraviolet radiation (UVA) Invest Ophthalmol Vis Sci 2010 51 1 129 138 10.1167/iovs.09-3738 19643975 PMC2869064

[B11] Kohlhaas M Spoerl E Schilde T Unger G Wittig C Pillunat LE Biomechanical evidence of the distribution of cross-links in corneastreated with riboflavin and ultraviolet A light J Cat Ref Surg 2006 32 2 279 283 10.1016/j.jcrs.2005.12.092 16565005

[B12] Spoerl E Huhle M Seiler T Induction of cross-links in corneal tissue Exp Eye Res 1998 66 1 97 103 10.1006/exer.1997.0410 9533835

[B13] Spörl E Huhle M Kasper M Seiler T Erhöhung der Festigkeit der Hornhaut durch Vernetzung Ophthtalmologe 1997 94 12 902 906 10.1007/s003470050219 9487761

[B14] Wollensak G Spoerl E Seiler T Stress–strain measurements of human and porcine corneas after riboflavin–ultraviolet-A-induced cross-linking J Cat Ref Surg 2003 29 9 1780 1785 10.1016/S0886-3350(03)00407-3 14522301

[B15] Spoerl E Wollensak G Seiler T Increased resistance of crosslinked cornea against enzymatic digestion Curr Eye Res 2004 29 1 35 40 10.1080/02713680490513182 15370365

[B16] Snibson GR Collagen cross-linking: a new treatment paradigm in corneal disease - a review Clin Experiment Ophthalmol 2010 38 2 141 153 10.1111/j.1442-9071.2010.02228.x 20398104

[B17] Muller L Thiel MA Kipfer-Kauer AI Kaufmann C Corneales Crosslinking als zusätzliche Therapieoption bei einschmelzender Hornhaut: eine Fall-Serie [Corneal cross-linking as supplementary treatment option in melting keratitis: a case series] Klin Monatsbl Augenh 2012 229 4 411 415 10.1055/s-0031-1299420 22496015

[B18] Corbin F Pathogen inactivation of blood components: current status and introduction of an approach using riboflavin as a photosensitizer Int J Hematol 2002 76 253 257 12430933 10.1007/BF03165125

[B19] Douki T Cadet J Modification of DNA bases by photosensitized one-electron oxidation Int J Radiat Biol 1999 75 5 571 581 10.1080/095530099140212 10374939

[B20] Tsugita A Okada Y Uehara K Photosensitized inactivation of ribonucleic acids in the presence of riboflavin Biochim Biophys Acta 1965 103 2 360 363 10.1016/0005-2787(65)90182-6 5319746

[B21] Makdoumi K Bäckman A Mortensen J Crafoord S Evaluation of antibacterial efficacy of photo-activated riboflavin using ultraviolet light (UVA) Graefes Arch Clin Exp Ophthalmol 2010 248 2 207 212 10.1007/s00417-009-1231-2 19921518

[B22] Martins SAR Combs JC Noguera G Camacho W Wittmann P Walther R Cano M Dick J Behrens A Antimicrobial efficacy of riboflavin/UVA combination (365 nm) in vitro for bacterial and fungal isolates: a potential New treatment for infectious keratitis Invest Ophthalmol Vis Sci 2008 49 8 3402 3408 10.1167/iovs.07-1592 18408193

[B23] Iseli HP Thiel MA Hafezi F Kampmeier J Seiler T Ultraviolet A/riboflavin corneal cross-linking for infectious keratitis associated with corneal melts Cornea 2008 27 5 590 594 10.1097/ICO.0b013e318169d698 18520510

[B24] Morén H Malmsjö M Mortensen J Öhrström A Riboflavin and ultraviolet A collagen crosslinking of the cornea for the treatment of keratitis Cornea 2010 29 1 102 104 10.1097/ICO.0b013e31819c4e43 19730094

[B25] Schnitzler E Spörl E Seiler T [Irradiation of cornea with ultraviolet light and riboflavin administration as a new treatment for erosive corneal processes, preliminary results in four patients] Klin Monatsbl Augenh 2000 217 3 190 193 10.1055/s-2000-10344 11076351

[B26] Makdoumi K Mortensen J Sorkhabi O Malmvall B-E Crafoord S UVA-riboflavin photochemical therapy of bacterial keratitis: a pilot study Graefes Arch Clin Exp Ophthalmol 2012 250 1 95 102 10.1007/s00417-011-1754-1 21874347

[B27] Makdoumi K Ultraviolet light (UVA) photoactivation of riboflavin as a potential therapy for infectious keratitis. PhD 2011 Örebro: Örebro University

[B28] Spoerl E Mrochen M Sliney D Trokel S Seiler T Safety of UVA-riboflavin cross-linking of the cornea Cornea 2007 26 4 385 389 10.1097/ICO.0b013e3180334f78 17457183

[B29] Dhaliwal JS Kaufman SC Corneal collagen cross-linking: a confocal, electron, and light microscopy study of eye bank corneas Cornea 2009 28 1 62 67 10.1097/ICO.0b013e31818225c3 19092408

[B30] Wollensak G Spoerl E Reber F Seiler T Keratocyte cytotoxicity of riboflavin//UVA-treatment in vitro Eye 2004 18 7 718 722 10.1038/sj.eye.6700751 14739922

[B31] Wollensak G Spoerl E Wilsch M Seiler T Keratocyte apoptosis after corneal collagen cross-linking using riboflavin/UVA treatment Cornea 2004 23 1 43 49 10.1097/00003226-200401000-00008 14701957

[B32] Wollensak G Spoerl E Wilsch M Seiler T Endothelial cell damage after riboflavin–ultraviolet-A treatment in the rabbit J Cat Ref Surg 2003 29 9 1786 1790 10.1016/S0886-3350(03)00343-2 14522302

[B33] Wollensak G Spoerl E Seiler T Riboflavin/ultraviolet-a–induced collagen crosslinking for the treatment of keratoconus Am J Ophthalmol 2003 135 5 620 627 10.1016/S0002-9394(02)02220-1 12719068

[B34] Wollensak G Iomdina E Dittert D-D Herbst H Wound healing in the rabbit cornea after corneal collagen cross-linking with riboflavin and UVA Cornea 2007 26 5 600 605 17525659 10.1097/ICO.0b013e318041f073

[B35] Mazzotta C Traversi C Baiocchi S Caporossi O Bovone C Sparano MC Balestrazzi A Caporossi A Corneal healing after riboflavin ultraviolet-A collagen cross-linking determined by confocal laser scanning microscopy in vivo: early and late modifications Am J Ophthalmol 2008 146 4 527 533 e521 10.1016/j.ajo.2008.05.042 18672225

[B36] Pot S Florin M Spiess B Hafezi F Treatment of "melting" ulcers in dogs and cats with UV-A/riboflavin cross-linking of corneal collagen (CXL): a pilot study Vet Ophthalmol 2011 14 4 281 10.1111/vop.12027 23356663

[B37] Berman MB Collagenase inhibitors: rationale for their Use in treating corneal ulceration Int Ophthalmol Clin 1975 15 4 49 66 10.1097/00004397-197501540-00006 57940

[B38] Brooks DE Ophthalmology for the equine practitioner 2002 Jackson, WY, USA: Teton NewMedia

[B39] Brooks DE Andrew SE Biros DJ Denis HM Cutler TJ Strubbe DT Gelatt KN Ulcerative keratitis caused by beta-hemolytic Streptococcus equi in 11 horses Vet Ophthalmol 2000 3 2–3 121 125 11397293 10.1046/j.1463-5224.2000.00120.x

[B40] Andrew SE Brooks DE Smith PJ Gelatt KN Chmielewski NT Whittaker CJG Equine ulcerative keratomycosis: visual outcome and ocular survival in 39 cases (1987–1996) Equine Vet J 1998 30 2 109 116 10.1111/j.2042-3306.1998.tb04469.x 9535066

[B41] Barnett KC Crispin SM Lavach JD Matthews AG Equine ophthalmology: an atlas & text 2004 2 Oxford, UK: Elsevier Ltd 98 125

[B42] Wollensak G Spörl E Reber F Pillunat L Funk R Corneal endothelial cytotoxicity of riboflavin/UVA treatment in vitro Ophthalmic Res 2003 35 6 324 328 10.1159/000074071 14688422

[B43] Kurtin WE Song P-S Photochemistry of the model phototropic system involving flavins and indoles - I. Fluorescence polarization and MO calculations of the direction of the electronic transition moments in flavins Photochem Photobiol 1968 7 3 263 273 10.1111/j.1751-1097.1968.tb08015.x 5648241

[B44] Goldich Y Marcovich AL Barkana Y Avni I Zadok D Safety of corneal collagen cross-linking with UV-a and riboflavin in progressive keratoconus Cornea 2010 29 4 409 411 20164744 10.1097/ICO.0b013e3181bd9f8c

[B45] Hovakimyan M Guthoff RF Stachs O Collagen cross-linking: current status and future directions J Ophthalmol 2012 article ID 406850 10.1155/2012/406850 PMC3263643 22288005

[B46] Koppen C Vryghem JC Gobin L Tassignon M-J Keratitis and corneal scarring after UVA/riboflavin cross-linking for keratoconus J Refract Surg 2009 25 9 S819 S823 10.3928/1081597X-20090813-11 19772258

[B47] Koller T Mrochen M Seiler T Complication and failure rates after corneal crosslinking J Cat Ref Surg 2009 35 8 1358 1362 10.1016/j.jcrs.2009.03.035 19631120

[B48] Burns FR Gray RD Paterson CA Inhibition of alkali-induced corneal ulceration and perforation by a thiol peptide Invest Ophthalmol Vis Sci 1990 31 1 107 114 2153643

[B49] Köhler A Ein neues Beleuchtungsverfahren fur mikrophotographische Zwecke Zeitschrift fur wissenschaftliche Mikroskopie und fur Microscopische Technik 1893 10 4 433 440

